# Supplementary material for: Correction of transposase sequence bias in ATAC-seq data with rule ensemble modeling
Source: NAR Genom Bioinform. 2023 Jun 2;5(2):lqad054. doi: 10.1093/nargab/lqad054 (PMC10236359; doi:10.1093/nargab/lqad054)
Supplement: lqad054_Supplemental_File [file lqad054_supplemental_file.pdf]

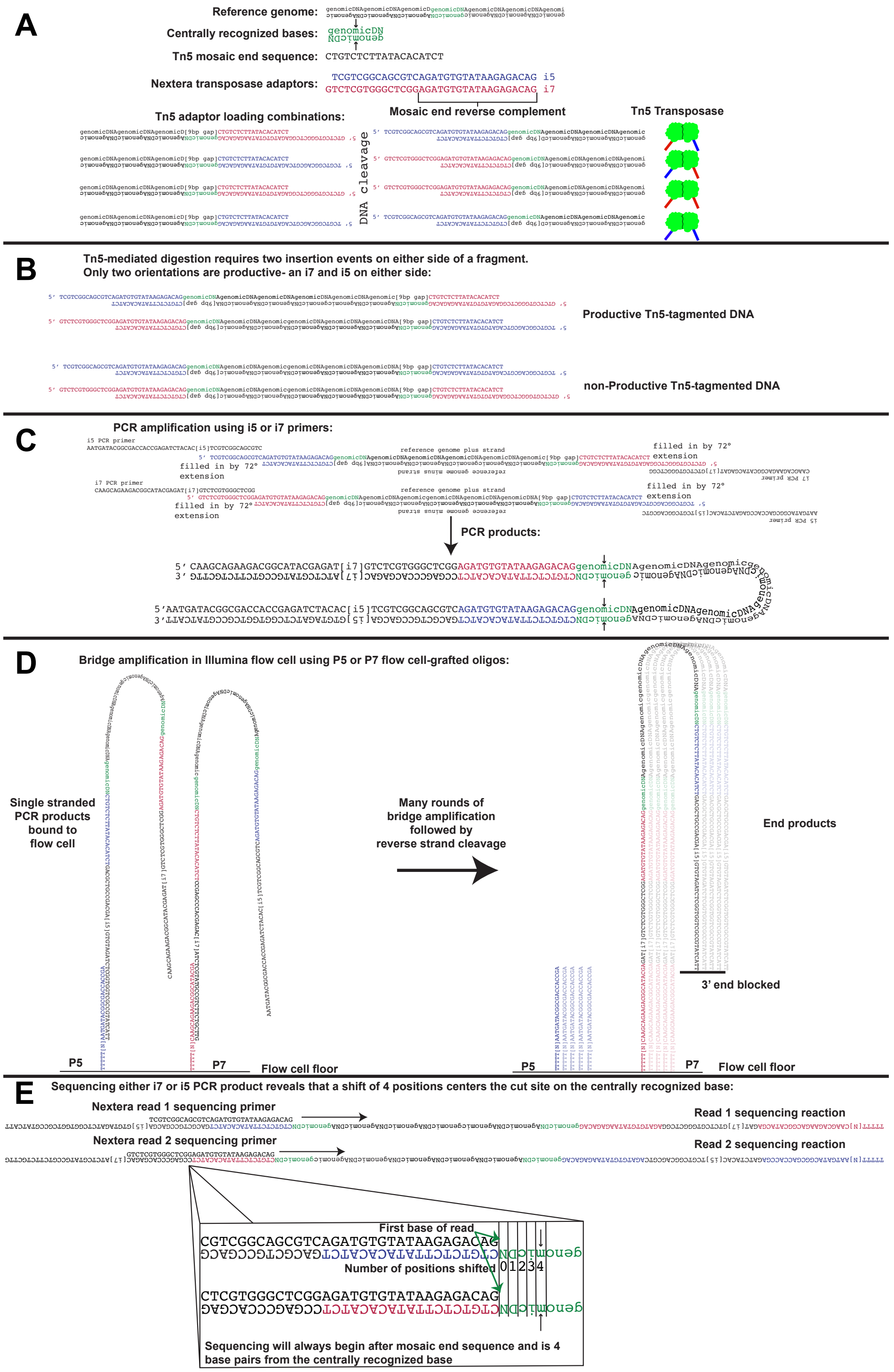

Fig. S1. Shifting forward and reverse aligned ATAC-seq data reads by 4 bases captures the center of the Tn5 duplication. A) Each monomer of the Tn5 transposase dimer is loaded with one of two Nextera adapter sequences. During an insertion event, each adapter is attached to one side of the duplicated 9 base pairs. Each adapter sequence is a concatenation of the Tn5 hyperactive mosaic end sequence and either an i7 or i5 sequencing primer. Each Tn5 dimer is capable of four possible DNA insertion events, based on which adapters are loaded into each Tn5 monomer. B) For a fragment to be sequenced, it must have an i7 and i5 primer on either side. Two possible combinations result in non-productive fragments. C) i5 and i7 overhangs are first filled in by a 72° extension step. This filled in sequence is then used as a template for PCR amplification of fragments. D) To begin bridge amplification, PCR products are first denatured into single strands. Next, these single strands are flowed over a lawn of oligos grafted to the flow cell floor which have a complementary sequence that anneals to the P5 or P7 sequences of the index 1 (i7) or index 2 (i5) PCR primers. Once annealed, complementary strands are then polymerized and the original binding strand washed away after being denatured. The single stranded, newly polymerized sequence is then able to bind to a complementary oligo grafted to the flow cell, and the complementary strand is polymerized and subsequently denatured. This process repeats many times to create clonal colonies of each read. Finally, the reverse strands are cleaved and washed away and 3′ ends of bound reads are blocked. The initial binding and end product stages of bridge amplification are depicted in the panel. E) Sequencing primers are used to determine the sequence downstream of the insertion event. Regardless of orientation, the first base of every read (the end of the adapter sequence) will be 4 positions away from the centrally recognized base.

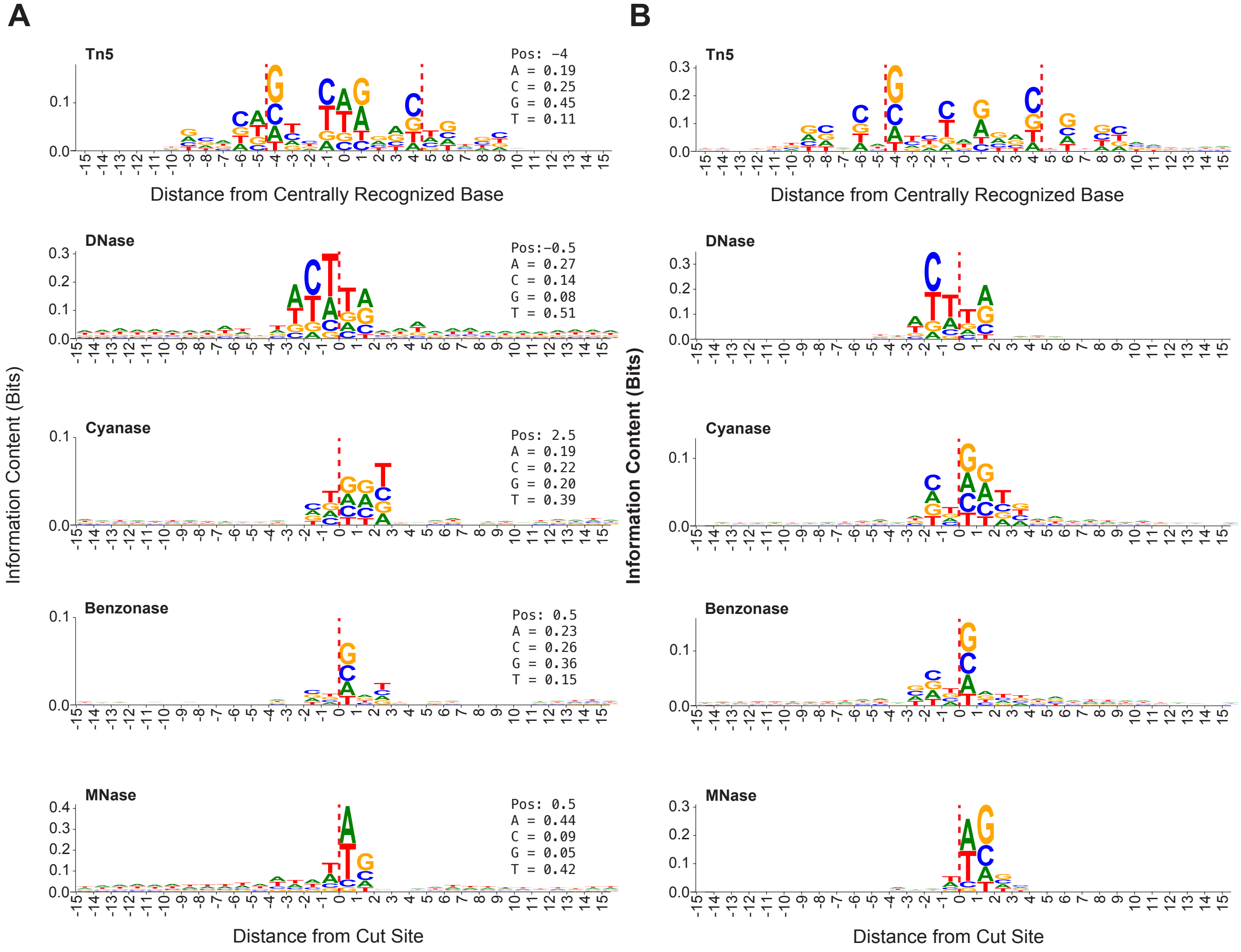

Fig. S2. Tn5 sequence bias motif is more complex than nucleases. A) These sequence logos (36, 45) representing enzyme biases were generated with equiprobable background nucleotide frequencies, which does not incorporate information about genomic AT/CG content. Nucleotide frequencies listed in the inset to the right correspond to the highest information content position (Pos). Nuclease cleavage sites are indicated by the dashed red line. B) Plots of enzymatic sequence bias motifs as seen in (Figure 1A), with the y-axis limit scaled to the position with highest information content.

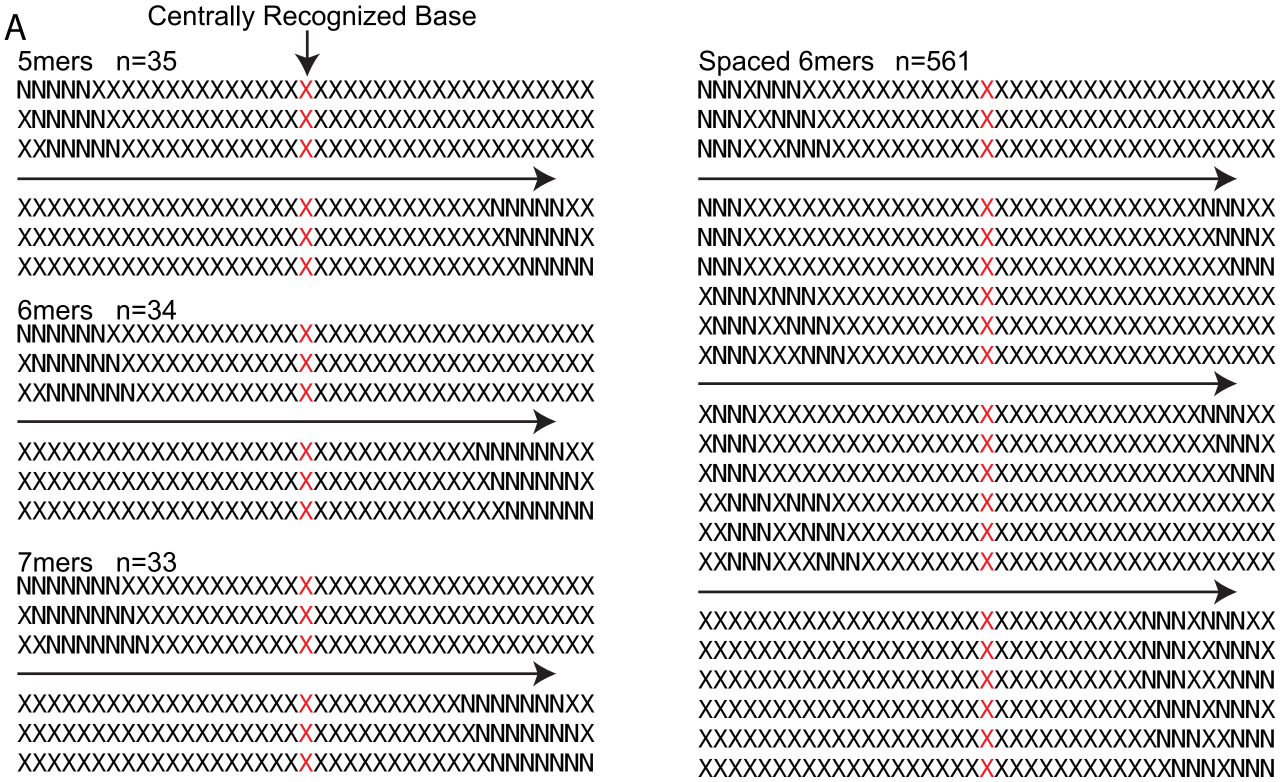

Fig. S3. Many 5/6/7-mer and spaced 6-mer combinations were inputs to rule ensemble modeling of Tn5 bias. A) This graphic depicts direct k-mer scaling positions used as input for the Tn5 rule ensemble model. In this example, the positions marked 'X' represent unmodeled positions, while the ones marked 'N' are the positions which each read is scaled by. The 'n' in the upper left corner is the number of permutations per k-mer size. Each graphic for each contiguous k-mer size represents the first three and last three input positions. The graphic for spaced 6-mer positions shows how the leading 3-mer is moved across the span, followed by a single base pair movement of the trailing 3-mer, to sample all possible combinations of spacing between the two.

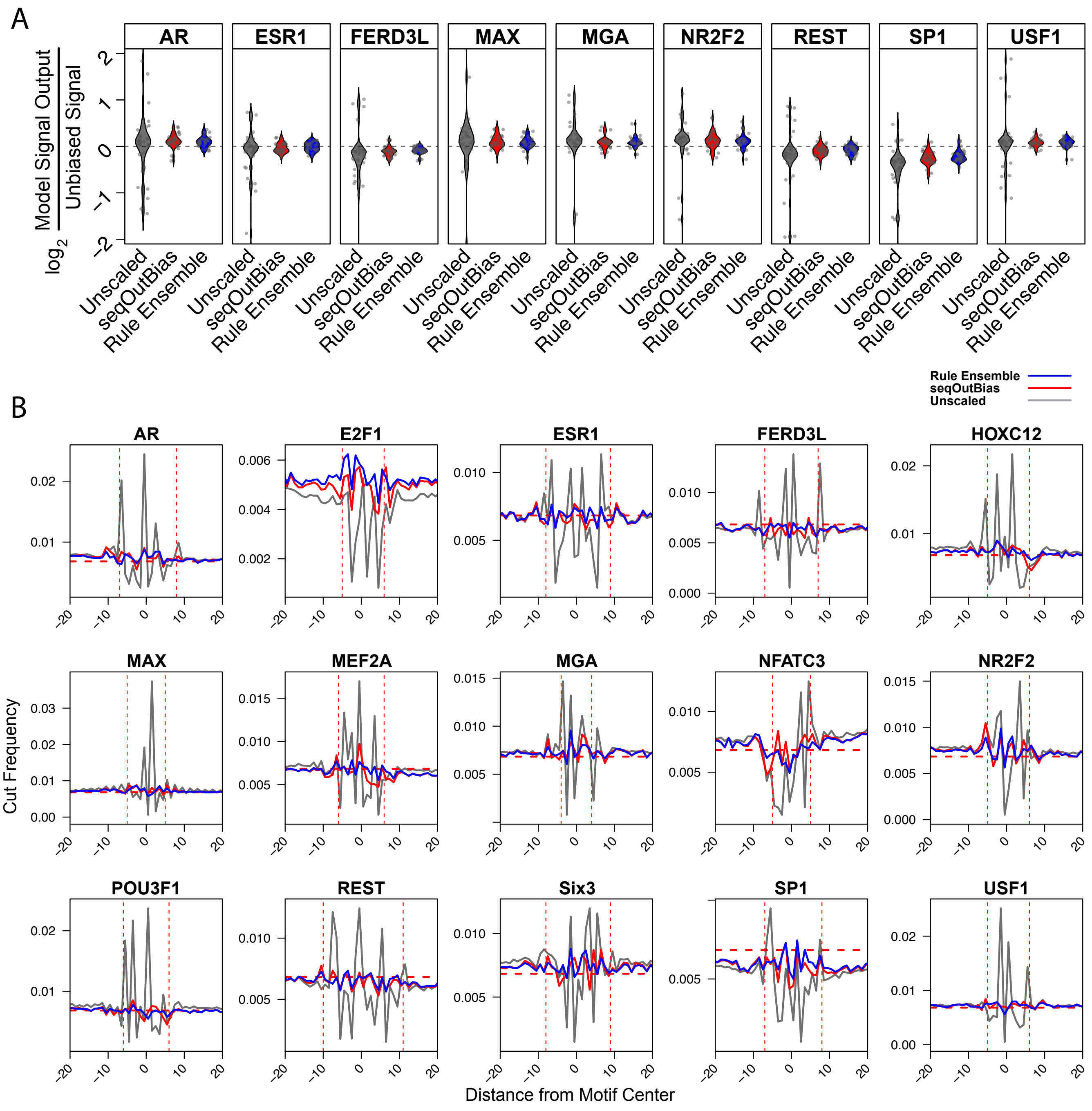

Fig. S4. Rule ensemble modeling corrects DNase bias. A) The remaining violin plots which compare the $\log_2$ of output divided by unbiased signal for each test transcription factor's composite profile. B) Composite profiles of the test set transcription factors show correction of DNase sequence bias. Each plot shows rule ensemble output compared with direct k-mer scaling of the 5-mer that encompasses the five most influential positions in Figure 5A. The horizontal dashed red lines indicate random cleavage, while the vertical dashed red lines indicate the beginning and end of the motif.

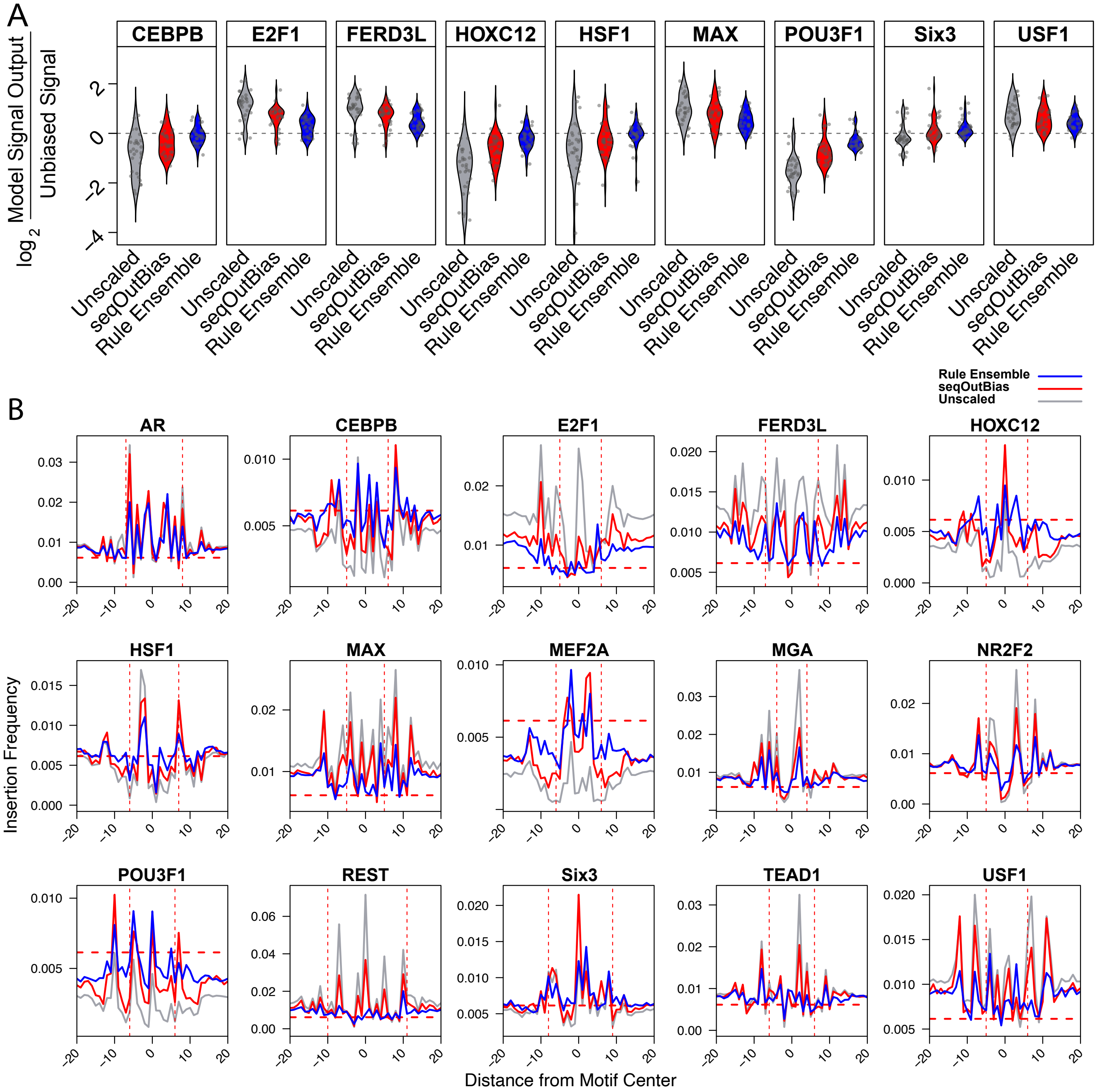

Fig. S5. Rule ensemble modeling corrects Tn5 bias. A) The single nucleotide bias correction comparison of the $\log_2$ ratio of model output to unbiased signal for each transcription factor indicate that rule ensemble outperforms direct k-mer scaling. These motifs represent the remaining test set motifs that are not in Figure 6. B) The composite profiles of transcription factors from rule ensemble output compared with direct k-mer scaling highlight the improvement of rule ensemble modeling. Dashed horizontal red lines denote random cleavage and the dashed vertical lines bookend each transcription factor motif.

Figure 5B summary statistics

Treatment	Abs Mean	Abs Variance	Unscaled Mann−Whitney U test p−value	seqOutBias Mann−Whitney U test p−value	Unscaled F−test p−value	seqOutBias F−test p−value
Unscaled	0.48	0.35	–	–	–	–
seqOutBias	0.17	0.019	***	–	***	–
Rule Ensemble	0.13	0.011	***	***	***	***

Table S1. Statistical tests for Figure 5B and Figure S4A highlight the improvement rule ensemble correction provides. The absolute value mean and variance were determined for all data of a given treatment of DNase single nucleotide bias correction, as displayed in Figure 5B and S4A. The smaller the absolute $\log_2$ values of variance and mean are, the better bias correction is performing. A value of 0 would indicate perfect bias correction. Q-Q plots indicated that these data are not normally distributed, therefore Mann-Whitney U tests were used to compare differences in mean. F-tests were used to show differences in variation between the values output from the scaling methods. P-values for U and F-tests are indicated by asterisks: ***:p<0.0005, **:p<0.005, *:p<0.05

Figure 6C summary statistics

Treatment	Abs Mean	Abs Variance	Unscaled Mann−Whitney U test p−value	seqOutBias Mann−Whitney U test p−value	Unscaled F−test p−value	seqOutBias F−test p−value
Unscaled	1.1	0.59	–	–	–	–
seqOutBias	0.72	0.24	***	–	***	–
Rule Ensemble	0.43	0.1	***	***	***	***

Table S2. Statistical analysis of the metrics in Figure 6C and Figure S5A highlight the improvement that rule ensemble modeling provides. For all motifs and positions within the motifs (±10bp), we $\log_2$ transformed the scaled values, converted to their absolute values, and reported the resultant means and variances of each scaling method from the data in Figure 6C and Figure S5A. The closer values are to 0 for each group, the more effective that treatment is at correcting Tn5 single nucleotide bias. The distribution of values for each method was not normal, so we used the Mann-Whitney U test for comparison. We compared the variances using an F-test. P-values for Mann-Whitney U and F-tests are indicated by asterisks. ***:p<0.0005, **:p<0.005, *:p<0.05

Figure 7D summary statistics

group	Abs Mean	Abs Variance	Unscaled t−test p−value	seqOutBias t−test p−value	Unscaled F−test p−value	seqOutBias F−test p−value
Unscaled	0.7	0.13	–	–	–	–
seqOutBias	0.52	0.078	***	–	0.12	–
Rule Ensemble	0.42	0.047	***	***	**	0.14

Table S3. Statistical analysis of the data in Figure 7D indicates that rule ensemble modeling provides statistically significant improvement of regional sequence biases. Mean and variance are reported for the absolute values of regional $\log_2$ Tn5 composite data displayed in Figure 7D. Perfect bias correction in this comparison is a value of 0 for both measurements. The data is normally distributed, so we performed t-tests to compare differences between means and F-tests were used to measure differences between variance. P-values for F- and t-test are indicated by asterisks. ***:p<0.0005, **:p<0.005, *:p<0.05
